# Putative interchromosomal rearrangements in the hexaploid wheat (*Triticum aestivum* L.) genotype ‘Chinese Spring’ revealed by gene locations on homoeologous chromosomes

**DOI:** 10.1186/s12862-015-0313-5

**Published:** 2015-03-11

**Authors:** Jian Ma, Jiri Stiller, Zhi Zheng, Yuming Wei, You-Liang Zheng, Guijun Yan, Jaroslav Doležel, Chunji Liu

**Affiliations:** Triticeae Research Institute, Sichuan Agricultural University, Wenjiang, Chengdu, 611130 China; CSIRO Agriculture Flagship, 306 Carmody Road, St Lucia, QLD 4067 Australia; School of Plant Biology, The University of Western Australia, Perth, WA 6009 Australia; Centre of the Region Haná for Biotechnological and Agricultural Research, Institute of Experimental Botany, Šlechtitelů 31, CZ-78371 Olomouc, Czech Republic

**Keywords:** Interchromosomal rearrangements, Wheat genome, Translocation, Comparative genomics, Chinese Spring

## Abstract

**Background:**

Chromosomal rearrangements are a major driving force in shaping genome during evolution. Previous studies show that translocated genes could undergo elevated rates of evolution and recombination frequencies around these genes can be altered. Based on the recently released genome sequences of *Triticum urartu, Aegilops tauschii*, *Brachypodium distachyon* and bread wheat, an analysis of interchromosomal translocations in the hexaploid wheat genotype ‘Chinese Spring’ (‘CS’) was conducted based on chromosome shotgun sequences from individual chromosome arms of this genotype.

**Results:**

A total of 720 genes representing putative interchromosomal rearrangements was identified. They were distributed across the 42 chromosome arms. About 59% of these translocated genes were those involved in the well-characterized translocations involving chromosomes 4A, 5A and 7B. The other 41% of the genes represent a large numbers of putative interchromosomal rearrangements which have not yet been described. The number of the putative translocation events in the D subgenome was about half of those presented in either the A or B subgenomes, which agreed well with that the times of interaction between the A and B subgenomes almost doubled that between either of them and the D subgenome.

**Conclusions:**

The possible existence of a large number of interchromosomal rearrangements detected in this study provide further evidence that caution should be taken when using synteny in ordering sequence contigs or in cloning genes in hexaploid wheat. The identification of these putative translocations in ‘CS’ also provide a base for a systematic evaluation of their presence or absence in the full spectrum of bread wheat and its close relatives, which could have significant implications in a wide array of fields ranging from studies of systematics and evolution to practical breeding.

**Electronic supplementary material:**

The online version of this article (doi:10.1186/s12862-015-0313-5) contains supplementary material, which is available to authorized users.

## Background

Chromosomal translocations are frequently associated with genomic disorders in human [1], animals [2] and microbes [3,4]. They are also a major driving force in shaping genome during evolution [1-5] and translocated genes could undergo elevated rates of evolution [2,6]. Results from previous studies also indicate that chromosomal translocations can alter levels of recombination and the chances of getting the desired recombinants will be diminished if the targeted gene is located near or at a translocation breakpoint [7].

The interchromosomal translocations involving chromosomes 4A, 5A and 7B in hexaploid wheat have been well documented [8-10]. The rearrangement between chromosomes 4A and 5A also exists in the diploid A genome donor of *Triticum urartu* [11] as well as in the diploid wheat *T. monococcum* [9], indicating that the 4/5 translocation predates the polyploidization event forming tetraploid wheat. Similarly, the translocation between chromosome 7B and the rearranged chromosome 4A appears to also exist in the tetraploid wheat [9], indicating that this translocation had occurred before the second polyploidization event which formed hexaploid wheat.

Several additional interchromosomal rearrangements have been described. One of these is a reciprocal translocation between chromosomes 5B and 7B which was very prevalent in West European wheats in the 1960s and 1970s [12] and is likely widespread in modern varieties [7]. Another example is the interchromosomal translocation between chromosomes 5B and 6B present in landraces of tetraploid wheat from Ethiopia [13]. It is of interest to note that both of these interchromosomal translocations seem to be present only in genotypes from specific geographical regions. Although it is not clear whether any of them is associated with modified morphological characteristics, their highly localized geographical distributions suggest that they could be associated with adaptability.

Previous studies on chromosomal translocation in wheat have been based on either cytology [14,15] or molecular markers [8,9]. These techniques have only limited resolutions which allow only the detection of rearrangements involving large chromosome segments. Recent progress in genome sequencing and single-copy FISH [16] offers the potential to drastically enhance the power of detecting chromosomal rearrangements in polyploidy wheats and their relatives. Based on the recently released genome sequences of *T. urartu* [17]*, Aegilops tauschii* [18], *B. distachyon* [19] and bread wheat [20], genes bordering each of the main translocation and inversion breakpoints on chromosomes 4A, 5A and 7B of the modern bread wheat genome were determined [21]. These genetic resources have also been exploited to assess interchromosomal rearrangements in the bread wheat genome and results obtained are reported here.

## Methods

### Data collection and analysis

Non-redundant orthologous gene sequences among CDSs (coding sequences) of *B. distachyon*, *T. urartu, Ae. tauschii* and wheat deletion bin-mapped ESTs (expressed-sequence tags) were identified in a recent study [22]. These sequences were analysed against the ‘CS’ shotgun sequences using the BLAST+ blastn algorithm with an E-value threshold of 10^−5^ (this value was applied in all subsequent BLAST analyses). The arm locations of genes on chromosome 3B were also identified previously [22]. For each of the non-redundant genes, the three best hits across the entire ‘CS’ genome were extracted. A gene was deemed to represent a putative interchromosomal rearrangement if any two of these three best hits were on different chromosomes from a given homoeologous group but the other one was on a chromosome belonging to a different homoeologous group [8,23]. For example, if the best three hits for a given gene were on chromosome arms 1AL, 1BL, and 2DS, the gene was considered to represent a putative translocation from 1DL to 2DS. For these genes, an additional 7 hits were then considered and the chromosomal locations were visually inspected again. Additional hits with different chromosomal locations from the first 3 hits were kept only. Only single-copy genes (the best 3 hits locating on the three chromosome arms belonging to a single homoeologous group) and those with simple duplications (3 of the best hits representing a single-copy genes and the other 3 hits representing a single homoeologous group, e.g. *Bradi2g55820.1*: 1AL, 1BL, 3BL, 2AL, 2BL, 2DL) were used in this study. For easy description, they were all classified as single-copy genes in this study. Genetic map locations of wheat contigs were obtained from http://www.wheatgenome.org/. [20]. A translocation was arbitrarily defined as the presence of at least two neighbouring genes with a maximum distance of 1.0 cM on at least one of the three wheat subgenomes.

### Configuration of chromosome 4A

The arm ratio of chromosome 4A was reversed due to translocations between chromosomes of 4A, 5A and 7B [8-10,24,25]. As suggested previously [22], we used ‘original 4AS’and ‘original 4AL’ to refer to the arms of the ancestral version of this chromosome and ‘modern 4AS’ and ‘modern 4AL’ to refer to the modern arm configuration of this chromosome.

### Validation of gene locations by PCR (polymerase chain reaction) amplification

Chromosome locations of a small number of genes identified from the above analysis were arbitrarily selected and validated using the euploid and nullisomic-tetrasomic and ditelosomic lines of ‘CS’. Annealing temperatures used ranges from 62 to 70°C depending on the primers (Additional file 1: Table S1). PCR products were separated on 1.5% agarose gels. If PCR products with similar sizes were generated from each of the wheat lines assessed, they were digested with a suitable restriction enzyme selected based on sequence alignments among the three subgenomes and then separated on gels.

## Results

**Figure 1:**
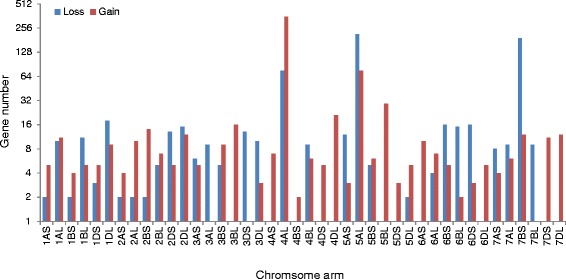
**Distribution of the 720 single-copy genes and genes with simple duplication representing interchromosomal rearrangements among the 42 chromosome arms of bread wheat.**

**Figure 2:**
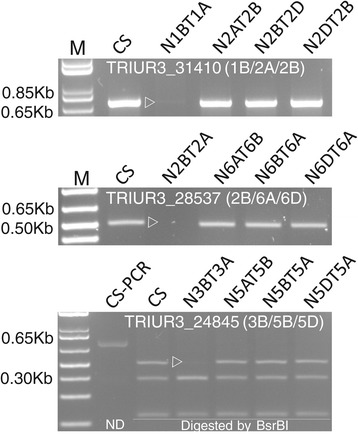
**PCR profiles of the hexaploid wheat genotype ‘Chinese Spring’ (‘CS’) nullisomic-tetrasomic (NT) lines with primers for 3 of the genes involved in putative interchromosomal rearrangements.** Locations of the genes detected from the chromosome shotgun sequences are given in brackets. 1Kb plus DNA ladder was used as the size marker (M). ND: non-digested control of the PCR product from ‘CS’. Fragments missing from the aneuploid lines of ‘CS’ are marked with open triangles.

A total of 720 single-copy genes representing interchromosomal rearrangements were detected. These genes were located on each of the 42 chromosome arms (Table 1, Figure 1). Map locations for about 64% of these genes on at least one of the three subgenomes are known (Additional file 2: Table S2). Seven of these genes were further assessed by PCR amplification against the euploid and nullisomic-tetrasomic lines of ‘CS’. Primers designed for two of these genes (*AEGTA13263*, *TRIUR3_25897*) failed to amplify. PCR products were successfully obtained for the other five genes and chromosome locations deduced from the chromosome shotgun sequences from the International Wheat Genome Sequencing Consortium (IWGSC) were confirmed for all of them (Figure 2).

**Table 1:** Locations of the 720 single-copy genes representing interchromosomal rearrangements on each of the 42 chromosome arms*

**Chr. arm**	**1AS**	**1AL**	**1BS**	**1BL**	**1DS**	**1DL**	**2AS**	**2AL**	**2BS**	**2BL**	**2DS**	**2DL**	**3AS**	**3AL**	**3BS**	**3BL**	**3DS**	**3DL**	**4AS**	**4AL**	**4BS**	**4BL**	**4DS**	**4DL**	**5AS**	**5AL**	**5BS**	**5BL**	**5DS**	**5DL**	**6AS**	**6AL**	**6BS**	**6BL**	**6DS**	**6DL**	**7AS**	**7AL**	**7BS**	**7BL**	**7DS**	**7DL**
1AS								1																							1											
1AL									1	2									1			1						5														
1BS								1																				1														
1BL								1	1			2	1			1				1			1					1								1			1			
1DS																1													1									1				
1DL																3												11			1					1					1	1
2AS																																						1				1
2AL																										1									1							
2BS			1																					1																		
2BL																1						1		1														2				
2DS			2	1											1						1			1		1			1	1	1		1								1	1
2DL		1											1			1		1		2				2				2		1	2	1									1	
3AS									1															1		1		1		2												
3AL						1						1																3					1	1		1	1					
3BS																			1					1	1							1	1									
3BL																								1																		
3DS									3		1								1			1	1	2			1		1						1							1
3DL		1			1	1		1				1							1	1							1					1										1
4AS							1																																			
4AL		1					1								1											71															1	
4BS															1																											
4BL		2				1				2		3					1																									
4DS																																										
4DL																																									1	
5AS	1					1	1	1			1					1			1	3		1															1					
5AL	1	3		1	2	1		1		2	2		2	1	1	4		1	1	167			2	4							1	1	1		1	1	2		9			2
5BS	1			2								1																										1				
5BL																																										
5DS																																										
5DL																			1	1																						
6AS																				1																						
6AL										1																															3	
6BS	1					1		1	3						1								1				2	2										1				3
6BL		1					1					1			2							1		1	2		1												2		3	
6DS					1			3	3						1					1	1			4																		2
6DL																																										
7AS					1								1									1		1				1		1	1	1										
7AL		1							2			1				1				1								1			1		1									
7BS	1		1	1		2					1					2				177				1		1	1	1			1	1		1								
7BL		1				1						2			1	1		1													1	1										
7DS																																				1						
7DL																																										

*Genes were transferred from chromosome arms in column to those in the row.

**Figure 3:**
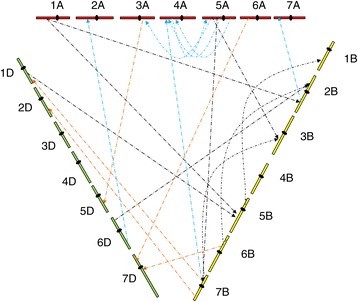
**Distribution of the 42 interchromosomal translocation events among chromosomes of the three bread wheat subgenomes.**

Based on the presence of at least two neighbouring genes with a map distance of no more than 1.0 cM in at least one of the three subgenomes, 21 groups of genes representing interchromosomal rearrangement were detected. The 42 events of interchromosomal rearrangement represented by these 21 groups of genes were located on 18 of the 21 wheat chromosomes: 17 in the A subgenome, 17 in the B subgenome and the other 8 in the D subgenome (Figure 3). These 42 translocation events involved a total of 443 genes. Map locations were known for 333 of them (or 75%) in the A subgenome, 213 (48%) in the B subgenome, and 303 (68%) in the D subgenome (Additional file 2: Table S2).

As expected, some of these translocations represent chromosome segments highly conserved between *Brachypodium* and wheat. One of the examples is represented by the two genes indicating a translocation from 1AL to 2BL (with homoeologous arm locations on 1BL, 1DL and 2BL, respectively). They are neighbouring genes in *Brachypodium* (*Bradi3g20170* and *Bradi3g20190*). They also have the same map locations on both 1BL (83.82 cM) and 2BL (82.72 cM), the only two chromosome arms on which their locations are available. Another example is the segment translocated from 1DL to 5BL (with homoeologous locations on 1AL, 1BL and 5BL, respectively) represented by *Bradi2g14170* and *Bradi2g14240* (Additional file 2: Table S2). However, some of the other groups representing interchromosomal translocations consist of genes with similar locations in wheat but apparently having originated from different *Brachypodium* chromosomes. One of these groups is the one representing a translocation from 1AL to 5BL (with chromosome arm locations on 1BL, 1DL and 5BL, respectively), which consists of genes from two separate *Brachypodium* chromosomes (Additional file 2: Table S2).

Not unexpectedly, the three dominating groups of genes representing interchromosomal translocations were those representing the well-known translocations involving chromosomes 4A, 5A and 7B. They consisted of 177, 167, 71 and 9 genes, respectively (Table 1), and in total accounted for about 59% of the 720 genes detected in this study. Apart from them, the group with the largest number of genes was the one representing a translocation from 1DL to 5BL (with homoeologous arm locations on 1AL, 1BL and 5BL, respectively). This group consists of 11 genes, which was even larger than those representing the 5AL segment on the modern 7BS (with chromosome arm locations on 5BL, 5DL and 7BS, respectively) (9 genes) resulted from the 4A/5A/7B translocations (Additional file 2: Table S2). Judged by their map locations, the 11 genes transferred from 1DL to the modern 5BL likely represent more than one translocation event. The terminal locations of *Bradi2g14170*, *Bradi2g14240* and *Bradi2g26795* infer a translocation from the terminal end of 1DL to the terminal end of the modern 5BL. However, the map locations of several others genes (including *Bradi2g26980*, *Bradi3g28810*, *TRIUR3_00282*, and *TRIUR3-27958*) indicate the existence of a second translocation involving an interstitially located segment from 1DL to 5BL (Additional file 2: Table S2). Each of the other groups representing interchromosomal translocations contained 4 or less of the single-copy genes used in this study. For those with known map locations, genes in each of the groups were all closely linked, indicating that each of these gene groups likely represented a single interchromosomal translocation event.

## Discussion

Taken advantage of the recently released chromosome arm-based sequences of the bread wheat genome, we conducted a systematic assessment of interchromosomal translocation in this important crop species. Even with the strict criteria used in this study, genes indicating the presence of interchromosomal rearrangement were detected on each of the 42 chromosome arms. About 59% of these genes were due to the well-known translocations involving chromosomes 4A, 5A and 7B. The other 41% of the translocated genes represent a large number of putative translocations scattered across chromosomes belonging to each of the three wheat subgenomes. Together with those putative intrachromosomal rearrangements reported earlier [22], the presence of such a large number of rearranged genes provides further evidence that caution should be taken when exploiting synteny in studying any of the three wheat genomes. As chromosomal translocations can alter levels of recombination and the chances of getting desired recombinants will be diminished if the gene conditioning the resistance is located near or at the translocation breakpoints [7], efficient breeding needs a clear understanding of how widespread these rearrangements exist in different wheat types and their close relatives.

For improving the likelihood that only genuine interchromosomal rearrangements were detected, only single-copy genes and those with simple duplications were selected for this study. The number of genes representing putative rearrangements could be significantly increased if the selection criteria were relaxed. For example, when the additional hits were tolerated, the number of genes representing the translocation from 6BS to 7DL would be increased from the currently 2 to 16. These additional genes with known map locations share a single location on either of the two non-translocated chromosome arms, 6AS or 6DS (Table 2), indicating that they likely represent a single chromosome segment on each of these two chromosomes. Thus it is not unreasonable to speculate that these genes could also represent a single segment on the third member of the homoeologous chromosome arm, 6BS, before the genes in concern were translocated to chromosome arm 7DL.

**Table 2:** **Genes representing a translocation from 6BS to 7DL**

**Gene name**	Locations*							
	**Details of the best 3 hits**						**Best 3 hits**	**Additional hits**
	**Contig_ID**	**Linkage (cM)**	**Contig_ID**	**Linkage (cM)**	**Contig_ID**	**Linkage# (cM)**		
Bradi1g35550.1	6AS_4384393	NA	6DS_2091402	81.58	7DL_3388333	NA	6AS,6DS,7DL	
Bradi3g06916.1	6AS_3107342	NA	6DS_2095270	81.58	7DL_3331018	111.08	6AS,6DS,7DL	
Bradi1g35592.1	6AS_4428500	60.96	6DS_2122226	81.58	7DL_3332022	NA	6AS,6DS,7DL	2DL,5DL
Bradi3g05560.1	6AS_4392917	60.96	6DS_2080416	81.58	7DL_3328281	142.97	6AS,6DS,7DL	1BS,3DL,4AL,7DS
Bradi3g05810.1	6AS_4403468	60.96	6DS_2098202	81.58	7DL_3393819	152.76	6AS,6DS,7DL	4AL,4DS,7AL
Bradi3g05960.1	6AS_4357746	60.96	6DS_2125465	81.58	7DL_3358080	160.68	6AS,6DS,7DL	5BL,5DL,7AL
Bradi3g07970.1	6AS_4351841	60.96	6DS_2058543	81.58	7DL_3349115	111.08	6AS,6DS,7DL	4AL,4BS,4DS,5BL,7BL
TRIUR3_05619	6AS_4354230	60.96	6DS_2082494	81.58	7DL_3392566	191.24	6AS,6DS,7DL	1AL,1BL,1DL,7DS
TRIUR3_15544	6AS_4357746	60.96	6DS_2125465	81.58	7DL_3358080	160.68	6AS,6DS,7DL	5BL,5DL,7AL
AEGTA43678	6AS_4359749	NA	6DS_2056711	81.58	7DL_3390442	NA	6AS,6DS,7DL	6DL,7DS
AEGTA43755	6AS_4365865	NA	6DS_2086887	81.58	7DL_3342428	120.28	6AS,6DS,7DL	3B,4AS,4DL,7BS,7DS
Bradi3g06670.1	6AS_4384393	NA	6DS_2091402	81.58	7DL_3388333	NA	6AS,6DS,7DL	5BL,5BS,5DL,5DS,6AL,6DL,7BS
Bradi3g16020.1	6AS_148467	NA	6DS_2077560	81.58	7DL_3361916	NA	6AS,6DS,7DL	4DL,5AL,5BL,7AL
Contig36859	6AS_4324849	NA	6DS_1084274	81.58	7DL_3331672	NA	6AS,6DS,7DL	1AL,1BL,2AL,2BL,5BL,5DL,6AL
Contig99362	6AS_148467	NA	6DS_2077560	81.58	7DL_3361916	NA	6AS,6DS,7DL	4DL,5AL,5BL,5DL
TRIUR3_17134	6AS_3958480	NA	6DS_2065488	81.58	7DL_3396087	160.28	6AS,6DS,7DL	1AL,1AS,1BS,2AL,2AS,7BL

*4AS and 4AL refer to the short and long arms, respectively, of the modern chromosome 4A.

*NA indicates map locations not available.

The genes selected for this study were likely to be significantly under-estimated for another three reasons: Firstly, genes which detected sequences on one or two of the three wheat subgenomes were not considered. As the genome sequences used in this study is known to be incomplete [20], additional genes meeting the selection criteria used will likely become available with the improved genome coverage. Secondly, single chromosome arms are the smallest unit resolved for most of the genes used in this study. Although more than one segment on a modern chromosome arm could having been translocated from different parts of the wheat genome [21], the limited numbers of single-copy genes used in this study and the lack of map locations for many of them (Additional file 2: Table S2) made it difficult to reliably identify multiple rearrangement events within a single chromosome arm. For the same reasons, reliable identification of genes immediately flanking the breakpoints to precisely characterize the majority of the putative rearrangements may have to wait. Thirdly, the lack of map locations for many of the selected genes (Additional file 2: Table S2) also hampered the selection of neighbouring genes used for declaring the presence of interchromosomal rearrangements. Thus, additional interchromosomal rearrangements are likely to be detected when map locations for more genes become available. Clearly, although synteny has been extensively and successfully used in many applications [26-29], the possible existence of such a high percentage of translocated genes suggest that caution need to be taken when applying this approach at the gene level.

Previous studies indicate that the D subgenome of wheat has several unique features. For example, molecular marker analysis has showed that the D subgenome is much less polymorphic than either the A or B subgenome [30,31]. Chromosome shotgun sequences of ‘CS’ showed that, among the three bread wheat subgenomes, the class I elements of transposons (retroelements) were the least abundant but the class II elements (DNA transposons) were the most abundant in the D subgenome [20]. The significantly smaller number of the putative interchromosomal translocation events detected in the D subgenome in this study seems to be another addition to the unique features of this subgenome. However, considering the A and B subgenomes joined together much earlier than the D subgenome in the two polyploidization events leading to the formation of the hexaploid wheat [20,32], the interchromosomal rearrangements seem to have occurred at a similar frequency among them.

Similar to those in other plant species, current systematics of wheat and its close relatives is based mainly on morphological characteristics. It is known that the principal differences between some of the species, such as those among the various hexaploid forms of wheat, are due to different alleles of one or two single genes [33]. Considering that translocations are often associated with significant disorders in various species including mammals, birds and bacteria [1-4], it is not unanticipated that changes due to these chromosomal rearrangements could be more drastic than those due to single genes in wheat as well. The identification of the putative interchromosomal translocations in this study and those intrachromosomal rearrangements in earlier studies [22,25,34,35] in the hexaploid wheat genotype ‘CS’ paved the way for a systematic assessment of their presence or absence across the full spectrum of bread wheat and its close relatives, which would not only allow the classification of bread wheat and its relatives on a more scientific basis but also facilitate the exploitation of genes from the wild relatives in wheat breeding programs.

## Conclusions

A total of 720 genes representing putative interchromosomal rearrangements in the bread wheat genotype ‘CS’ was detected in this study and the number of the genes was likely significantly underestimated. These genes were distributed on each of the 42 chromosome arms and they suggested the presence of a large number of putative interchromosomal rearrangements in this genotype. Together with those intrachromosomal rearrangements reported in earlier studies, these results provide further evidence showing that extensive structural differences likely exist among the three subgenomes of ‘CS’. An effort is urgently required to clarify which of these rearrangements were induced during the production of the aneuploids used for generating the shotgun sequences and which were specific to the genotype ‘CS’. A clear understanding of the presence or absence of these chromosomal rearrangements in the full spectrum of bread wheat and its close relatives could dramatically improve our capacity in wheat genome research as well as in new variety breeding.

## Additional files

Additional file 1: Table S1. Primer sequences used for validating chromosomal locations of selected genes. Additional file 2: Table S2. Details of the 720 Single-copy genes representing possible interchromosomal rearrangements in wheat.

## Abbreviations

CDS: Coding sequence
CS: Chinese Spring
IWGSC: The international wheat genome sequencing consortium
EST: Expressed-sequence tag
PCR: Polymerase chain reaction
